# Risk of interference between the tibial tunnel and locking screws in medial meniscus posterior root repair and open wedge high tibial osteotomy

**DOI:** 10.1186/s40634-022-00464-0

**Published:** 2022-03-15

**Authors:** Shuntaro Nejima, Ken Kumagai, Shunsuke Yamada, Masaichi Sotozawa, Dan Kumagai, Hironori Yamane, Yutaka Inaba

**Affiliations:** Department of Orthopaedic Surgery, Yokohama City University School of Medicine, 3-9 Fukuura, Kanazawa-ku, Yokohama, 236-0004 Japan

**Keywords:** Open wedge high tibial osteotomy, Transtibial pull-out repair, Medial meniscus posterior root tear

## Abstract

**Purpose:**

To evaluate whether the frequency of interference between locking screws for the plate fixation and tibial tunnels differs depending on the tibial tunnel positions in a surgical simulation of the transtibial pull-out repair of medial meniscus posterior root tears (MMPRTs) in patients undergoing biplanar open wedge high tibial osteotomy (OWHTO).

**Methods:**

Sixty-five patients (75 knees) who underwent OWHTO with TomoFix small plate (Depuy Synthes, PA, USA) for medial knee osteoarthritis with varus malalignment were enrolled in this study. Surgical simulation of transtibial pull-out repair of MMPRTs was performed using postoperative computed tomography images. The tibial tunnel was created in the anatomical attachment area of the medial meniscus posterior root. Another aperture of the tibial tunnel was created on the anteromedial (AM) tibial cortex, the posteromedial (PM) tibial cortex, and the anterolateral (AL) tibial cortex in the proximal tibial fragment. The frequency of interference between the tibial tunnel and A–D locking screws was compared in the 3 tibial tunnel positions. In each tibial tunnel position, the locking plate position with and without interference between the tibial tunnel and at least one locking screw was compared.

**Results:**

For screw A, the frequency of interference with the tibial tunnel in the AL position was higher than that in the AM (*P* = 0.048) and PM positions (*P* <  0.001). For screws B and C, the frequency of interference with the tibial tunnel in the AM position was higher than that in the PM (*P* <  0.001, *P* = 0.007) and AL positions (*P* <  0.001, *P* = 0.001), respectively. For screw D, there was no difference in the frequency of interference with the tibial tunnel among the three positions. The frequency of interference between the tibial tunnel and at least one screw in the AM position was 100%, which was higher than that in the PM (*P* <  0.001) and AL positions (*P* <  0.001). In the PM position, the locking plate was placed more posteriorly in the group where the locking screw interfered with the tibial tunnel. In the AL position, the locking plate was placed more parallel to the medial/lateral axis of the tibial plateau in the interference group.

**Conclusion:**

Making the tibial tunnel in the AM position should be avoided because interference with locking screws was inevitable. When the tibial tunnel is created in the PM position, interference between the tibial tunnel and screw C should be paid attention. Anterior placement of the locking plate could be useful to prevent interference between locking screws and the tibial tunnel in the PM position. In addition, when the tibial tunnel is created in the AL position, interference between the tibial tunnel and especially screw A among screws A–C should be paid attention. Placing the locking plate in an anteromedial direction could be useful to prevent interference between locking screws and the tibial tunnel in the AL position.

**Level of evidence:**

IV

## Introduction

The medial meniscus posterior root plays important roles in anchoring the medial meniscus to its tibial attachment site and maintaining hoop stress mechanism. The contact pressure of the medial compartment of the knee after medial meniscus posterior root tears (MMPRTs) is higher than that of intact knees and the same as that after total meniscectomy [1]. MMPRTs lead to extrusion of the medial meniscus and consequent arthritic changes in the medial compartment of the knee [5, 13, 19, 23]. Transtibial pull-out repair of the medial meniscus is a surgical procedure for MMPRTs, and good clinical outcomes have been reported [2, 8, 10, 14, 17, 20, 24].

In knees with MMPRTs and varus malalignment, repair of MMPRTs with open wedge high tibial osteotomy (OWHTO) is a surgical option [6, 7, 11, 15, 18, 26] because isolated repair of MMPRTs with varus alignment of > 5° leads to poor clinical outcomes [3, 20]. Lee et al. [15] reported that the healing rate of MMPRTs after transtibial pull-out repair with OWHTO was better than that after isolated OWHTO. However, interference between locking screws and tibial tunnels could lead to suture damage in pull-out repair. Further, insufficient screw insertion could lead to inferior stability of plate fixation. There are three options for tibial tunnel positioning: anteromedial tibial cortex, posteromedial tibial cortex, and anterolateral tibial cortex. It is unclear which tunnel position is suitable to prevent interference of locking screws and the tibial tunnel.

The purpose of this study was to simulate to create the tibial tunnels for pull-out repair in CT images after OWHTO and evaluate the frequency of interference between locking screws and each tibial tunnel position. It was hypothesized that making the tibial tunnel in anteromedial tibial cortex led to higher rate of interference between screws and tibial tunnel compared to that in posteromedial and anterolateral tibial cortex because the tibial tunnel must pass between the proximal locking screws when the tibial tunnel is created from the anatomical attachment area of the medial meniscus posterior root to the anteromedial tibial cortex.

## Materials and methods

This study was approved by the ethics committee of our hospital (F210800005), and written informed consent was obtained from each patient.

A total of 78 patients (97 knees) who underwent biplanar OWHTO with TomoFix small plate (Depuy Synthes, PA, USA) for medial knee osteoarthritis (OA) with varus malalignment from June 2012 to September 2016 were enrolled. Any medial meniscus posterior root repairs were not performed in these patients. Computed tomography (CT) images were obtained postoperatively. Patients with OA of the hip (2 knees), a history of surgical treatment of the lower limbs (11 knees) or concomitant procedures (tibial tubercle transfer) (1 knee) were excluded. Knees with additional screws for type 3 lateral hinge fractures [27] or without D screws because the transverse cut was too high were excluded (2 knees). In addition, 3 knees were excluded because the locking plate was placed anteriorly and there was no space to create the tibial tunnel on the anteromedial side of the tibia. Moreover, 3 knees were excluded because the proximal tibial fragment was small and there is no space to create the tibial tunnel at the anterolateral side of the tibia. Thus, 65 patients (75 knees) met the inclusion criteria for this study. The demographic data are shown in Table 1.

**Table 1:** Patients’ demographic characteristics

Knees, n	75
Age, y	65.5 ± 7.9 (45–80)
Height, cm	157.6 ± 8.0 (138.5–182.9)
Weight, kg	63.8 ± 10.6 (43.8–92.4)
Body mass index, kg/m^2^	25.6 ± 3.5 (19.0–35.3)
Side, left/right	32/43
Gender, female/male	56/19
Ahlbӓck grade 1/2/3	59/12/4
Opening gap, mm	12.7 ± 2.7 (5.0–21.0)
Preoperative mMPTA, °	84.6 ± 1.8 (78.0–89.0)
Postoperative mMPTA, °	96.3 ± 2.5 (91.0–102.0)

Data are presented as means ± standard deviation with the range in parentheses. The mMPTA, mechanical medial proximal tibial angle

### Surgical procedure of OWHTO

The surgical planning was performed so that postoperative weight bearing line ratio was 62% using preoperative anteroposterior whole-leg standing radiographs. Two Kirschner wires were inserted 35 mm below the medial tibial plateau towards the tip of the fibular head under fluoroscopy. Transverse cutting was performed across the bottom of the wires using a bone saw and chisel, leaving the lateral cortex intact as a hinge. A 15-mm thickness of the tuberosity was left as the flange, and an ascending cut was performed at 100°–120° to the transverse cut. After the osteotomy site was opened as planned, one or two formed trapezoid β-TCP wedges (Olympus Terumo Biomaterials, Tokyo, Japan) were inserted into the opening gap. Then, the osteotomy site was fixed with a TomoFix small plate and 8 locking screws (Depuy Synthes, PA, USA).

### Surgical simulation of transtibial pull-out repair with OWHTO on CT images

**Fig. 1:**
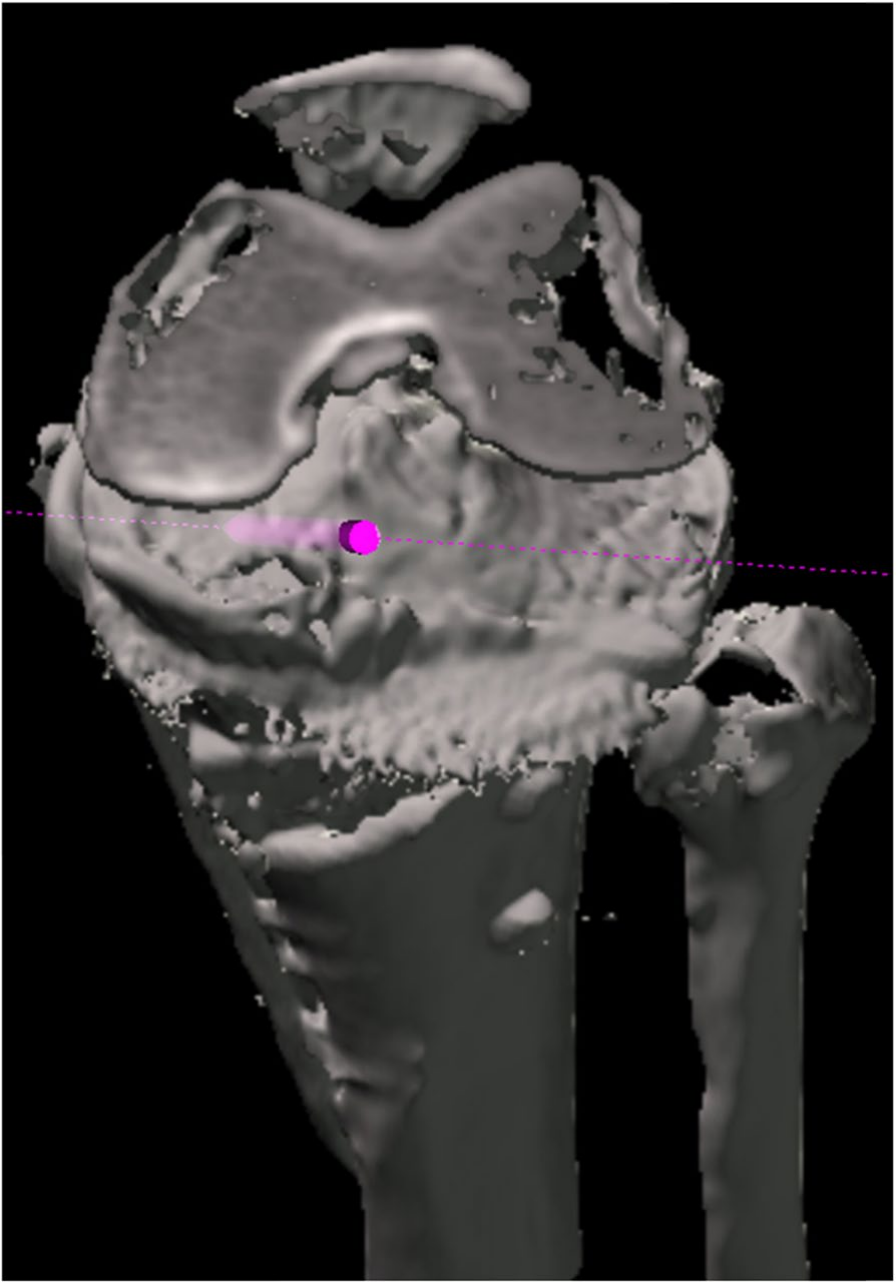
The tibial tunnel was made in the centre of the anatomical attachment area of the medial meniscus posterior root, which contacted three sides: the anterior border of the posterior cruciate ligament tibial attachment, lateral margin of the medial tibial plateau, and retro-eminence ridge [4]

**Fig. 2:**
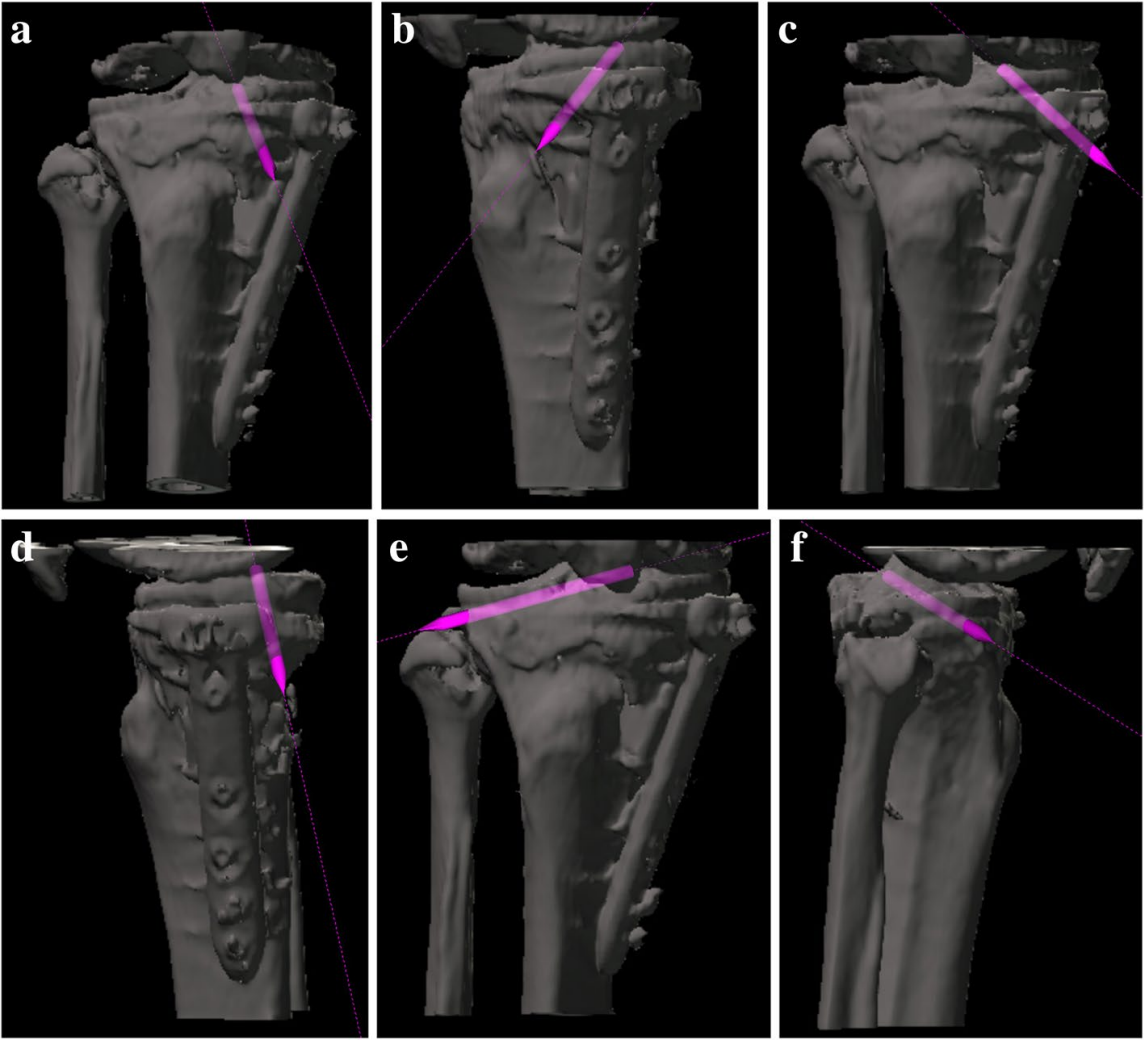
Another aperture of the tibial tunnel was made on the anteromedial tibial cortex positioned anterior to the locking plate (**a**, **b**), posteromedial tibial cortex positioned posterior to the locking plate (**c**, **d**), and anterolateral tibial cortex in the proximal tibial fragment (**e**, **f**). In the anterolateral position, planes of transverse and ascending cuts were made, and a tibial tunnel was made to fit into the proximal tibial fragment

**Fig. 3:**
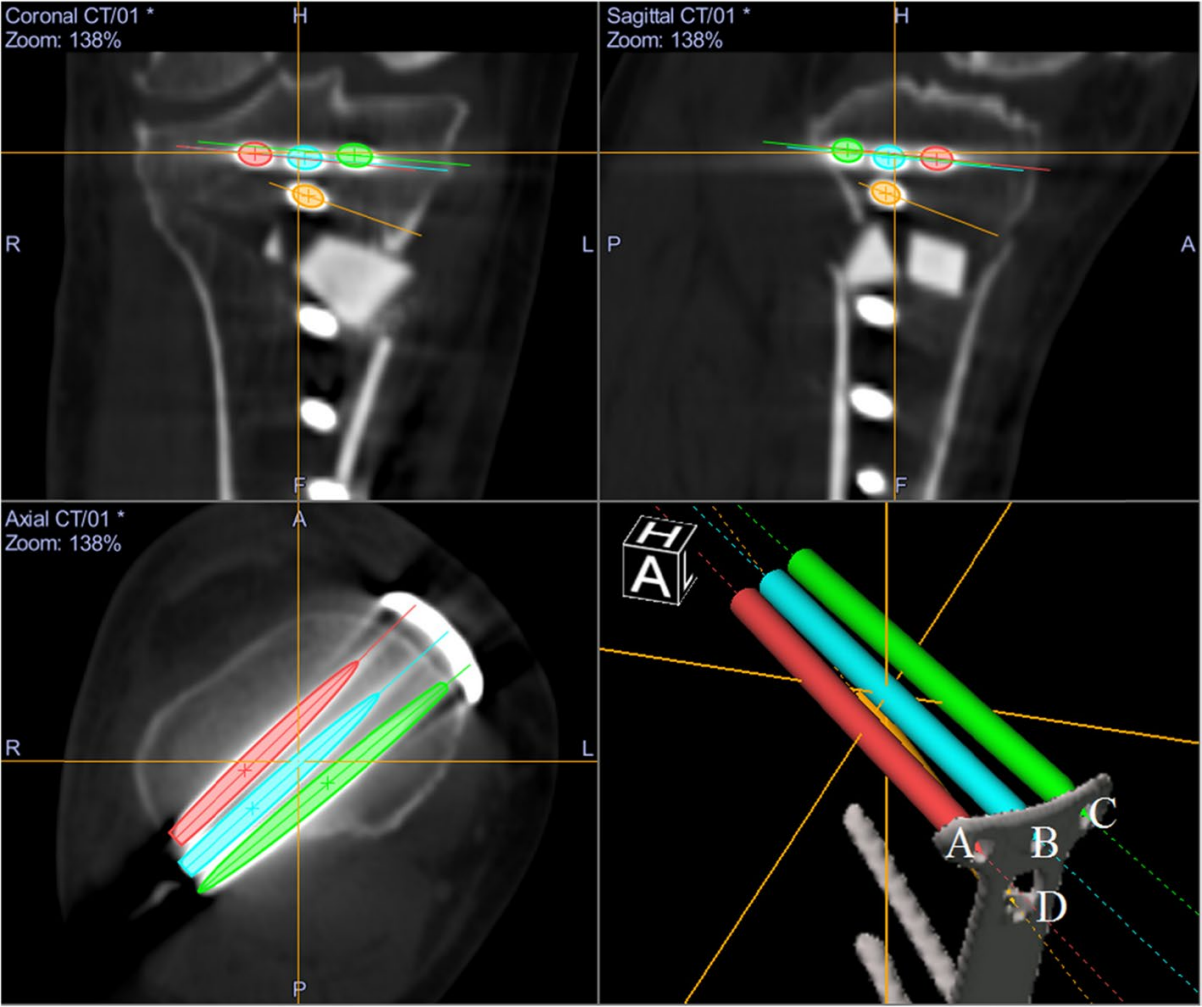
To avoid halation of locking screws on the CT image, another cylinder was made so that the centre of the cylinder passed the centre of the screw head and the tip of the screw. Four locking screws for the proximal tibial fragment were named A–D screws (**A** Proximal anterior screw; **B** Proximal middle; **C** Proximal posterior; **D** Distal)

**Fig. 4:**
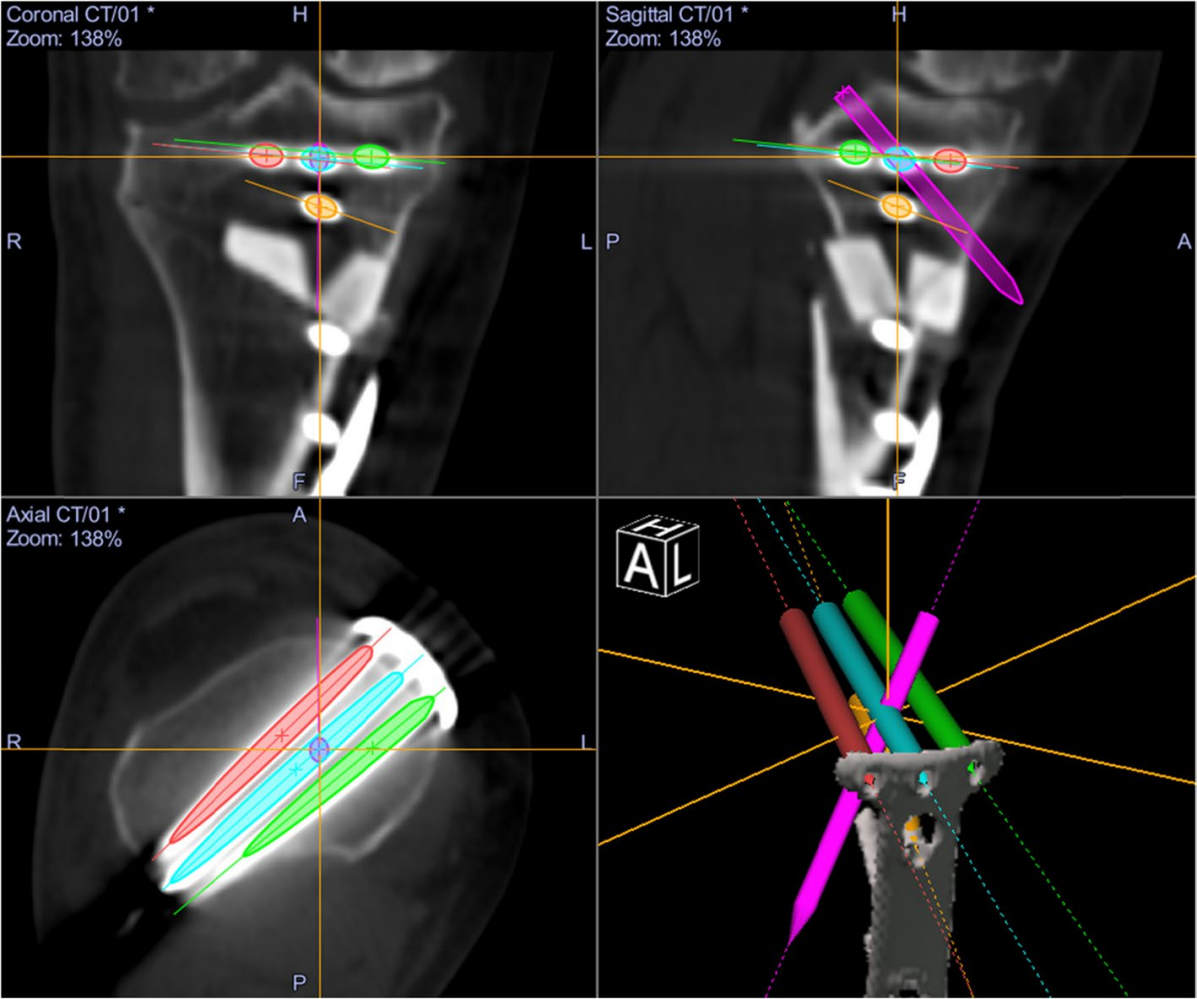
Each tibial tunnel was created in such a way that the number of screws interfering with the tibial tunnel was minimized. When the number of interfering screws was the same, the tibial tunnel positioning where the screw could be inserted the longest was chosen. In this case, the B screw interfered with the tibial tunnel, which was made on the anteromedial tibial cortex

Whole lower limb CT images (1.5-mm-thick slices) were obtained with the patients lying supine on a SOMATOM Sensation 16 scanner (Siemens, Munich, Germany). The data were imported into Orthomap 3D (Stryker, Kalamazoo, MI), which enabled the selection of anatomical landmarks and the measurement of three-dimensional linear and angular parameters by simultaneously showing the sagittal, coronal, and axial planes [12]. On postoperative CT images, surgical simulation of transtibial pull-out repair of medial meniscus posterior root tears was performed. The tibial tunnel was a cylindrical shape made in the centre of the anatomical attachment area of the medial meniscus posterior root, which contacted three sides: the anterior border of the posterior cruciate ligament tibial attachment, lateral margin of the medial tibial plateau, and retro-eminence ridge (Fig. 1) [4]. Another aperture of the tibial tunnel was made on the anteromedial (AM) tibial cortex positioned anterior to the locking plate (Fig. 2a, b), posteromedial (PM) tibial cortex positioned posterior to the locking plate (Fig. 2c, d), and anterolateral (AL) tibial cortex in the proximal tibial fragment. The AM position was defined so that the tibial tunnel was created between the medial edge of the flange and the locking plate. The PM position was defined so that the tibial tunnel was created between the locking plate and the medial edge of the posterior tibial cortex. The AL position was defined so that the tibial tunnel was created between the lateral edge of the flange and the fibular head. The posterolateral position was not defined because the tibial tunnel could not be created due to the fibular head. In the AL position, planes of transverse and ascending cuts were made, and a tibial tunnel was made to fit into the proximal tibial fragment (Fig. 2e, f). The diameter of the tibial tunnel was defined as 4.0 mm because the diameter of one of the reamers used for making the tibial tunnel was 4.0 mm (Arthrex, Naples, FL, USA). In addition, the tibial tunnel was made not to overlap with a locking plate. To avoid halation of locking screws on the CT image, another cylinder was made so that its centre passed the centre of the screw head and the tip of the screw. The diameter of this cylinder was defined as 5.0 mm because the diameter of the locking screw of the TomoFix system was 5.0 mm (Fig. 3). Four locking screws for the proximal tibial fragment were named A–D screws (A: proximal anterior screw; B: proximal middle; C: proximal posterior; D: distal) (Fig. 3). Each tibial tunnel was created in such a way that the number of screws interfering with the tibial tunnel was minimized. When the number of interfering screws was the same, the tibial tunnel positioning where the screw could be inserted the longest was chosen (Fig. 4).

### Evaluation of the locking plate position

**Fig. 5:**
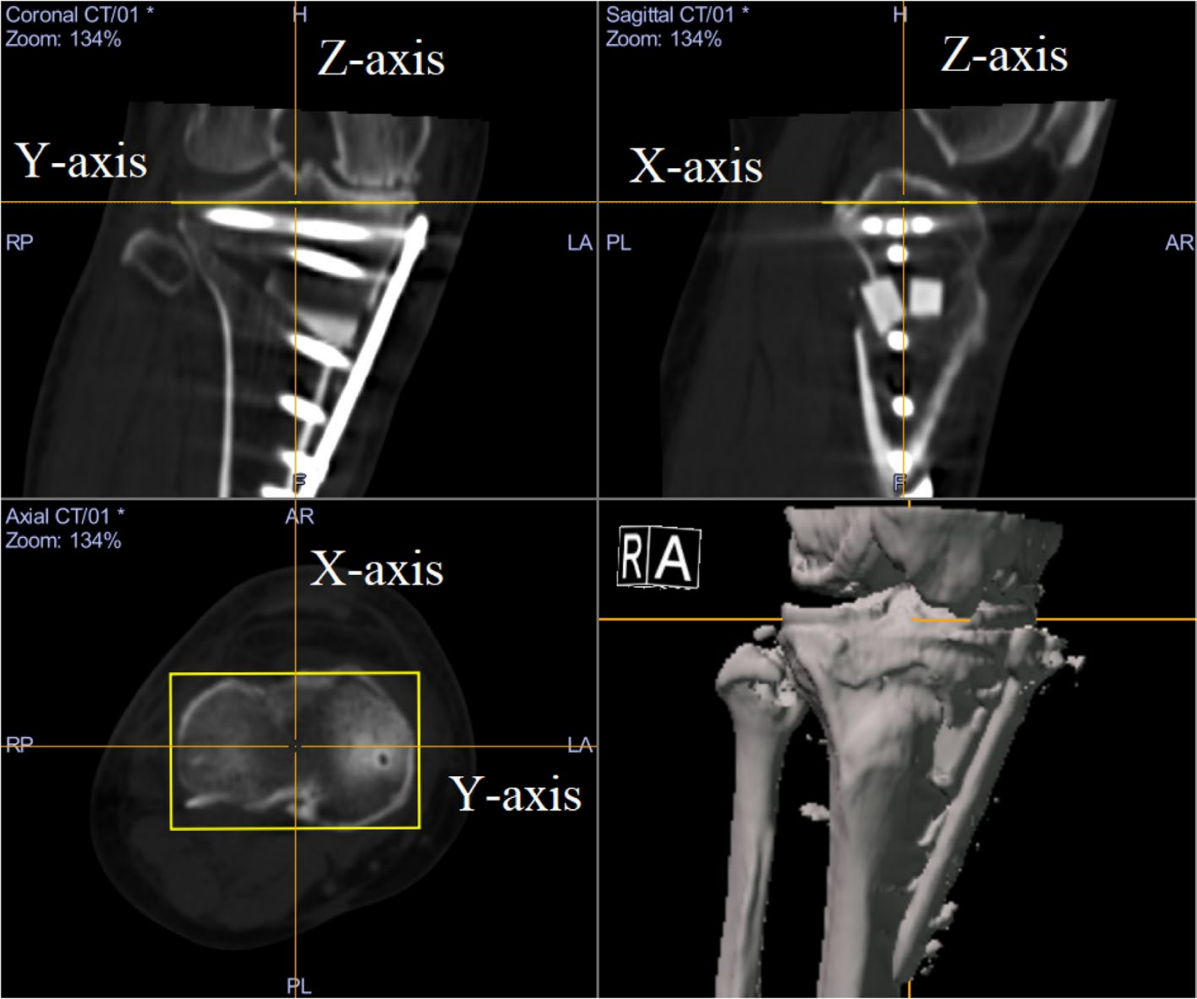
To evaluate the locking plate position on postoperative CT images, the coordinate system was defined around a virtual rectangle fitted onto the contour of the tibial plateau at the level above the proximal end of the fibula parallel to the tibial plateau. Anteroposterior, medial/lateral, and vertical axes was defined as the X-axis, Y-axis and, Z-axis, respectively

The locking plate position was evaluated on postoperative CT images. The coordinate system was defined around a virtual rectangle fitted onto the contour of the tibial plateau at the level above the proximal end of the fibula parallel to the tibial plateau. Anteroposterior, medial/lateral, and vertical axes was defined as the X-axis, Y-axis and, Z-axis, respectively (Fig. 5). The height of the locking plate position was defined as the distance between the tibial plateau and the centre of the most proximal point of the locking plate was measured according with the Z-axis. The anteroposterior locking plate position was defined as the distance between the most anterior point of the tibial tuberosity and the centre of screw B head according with the X-axis. In addition, the locking plate angle was defined as the angle between the Y-axis and the central axis of screw B.

### Statistical analysis

Continuous variables are expressed as means and standard deviations and normal distribution was confirmed using the Shapiro–Wilk test. The frequency of interference of the tibial tunnel and A–D screws was compared between 3 tibial tunnel positions using the Cochran-Q test which is a non-parametric way to find differences in matched sets of three proportions. If statistical significance was found in the comparison of the three tibial tunnel positions, pairwise comparisons between each of two tibial tunnel positions were performed and corrected for multiplicity using the Bonferroni test. In addition, the frequency of interference between the tibial tunnel and at least one screw was compared among the 3 tibial tunnel positions using the same statistical method. In each tibial tunnel position, the locking plate position with and without interference between the tibial tunnel and at least one locking screw was compared by Student’s t-test. All statistical analyses were performed using IBM SPSS for Windows, version 27.0 (IBM Corporation, Armonk, NY, USA). *P* values less than 0.05 were considered significant. A power analysis was performed on the Cochran-Q test (significance level = 0.05, sample size = 75). A post hoc power analysis resulted in a power of 0.8 to detect a 20% difference in the frequency of interference between the tibial tunnel and screws.

## Results

The frequency of interference of the tibial tunnel and each screw is shown in Table 2. For screw A, the frequency of interference with the tibial tunnel in the AL position was higher than that in the AM and PM position. For screws B and C, the frequency of interference with the tibial tunnel in the AM position was higher than that in the PM and AL position. For screw D, there was no difference in the frequency of interference with the tibial tunnel among the three tibial tunnel positions. The frequency of interference between the tibial tunnel and at least one screw in the AM position was 100% and higher than that in the PM and AL position.

**Table 2:** The frequency of interference between screws and each tibial tunnel position, n (%)

	Tibial tunnel position			*P* value for all group	*P* value for pairwise comparison		
	AM	PM	AL		AM vs PM	AM vs AL	PM vs AL
Screw A	6 (8.0%)	0 (0%)	15 (20.0%)	< 0.001	n.s.	0.048	< 0.001
Screw B	63 (84.0%)	0 (0%)	5 (6.7%)	< 0.001	< 0.001	< 0.001	n.s.
Screw C	20 (26.7%)	7 (9.3%)	5 (6.7%)	0.001	0.007	0.001	n.s.
Screw D	2 (2.7%)	0 (0%)	0 (0%)	n.s.	–	–	–
At least one screw	75 (100%)	7 (9.3%)	21 (28.0%)	< 0.001	< 0.001	< 0.001	n.s.

*AM* Anteromedial, *PM* Posteromedial, *AL* Anterolateral

The locking plate position with and without interference between the tibial tunnel and at least one locking screw is shown in Table 3. In the PM position, the locking plate was placed more posteriorly in the group where the locking screw interfered with the tibial tunnel. In the AL position, the locking plate was placed more parallel to the medial/lateral axis of the tibial plateau in the interference group.

**Table 3:** Locking plate position with and without interference between the tibial tunnel and at least one locking screw

Posteromedial position				
Variables	Total sample (*n* = 75)	Interference (*n* = 7)	Not interference (*n* = 68)	*P* value
Height of the locking plate position, mm	7.2 ± 2.5 (1.0–12.8)	7.1 ± 3.0 (3.2–10.8)	7.2 ± 2.4 (1.0–12.8)	n.s.
Anteroposterior locking plate position, mm	11.5 ± 6.4 (−1.4–25.2)	17.9 ± 5.4 (10.0–23.6)	10.8 ± 6.2 (− 1.4–25.2)	< 0.01
Locking plate angle,°	29.6 ± 10.0 (3.0–52.4)	25.3 ± 9.8 (15.1–39.0)	30.1 ± 10.0 (3.0–52.4)	n.s.
Anterolateral position				
Variables	Total sample (*n* = 75)	Interference (*n* = 21)	Not interference (*n* = 54)	*P* value
Height of the locking plate position, mm	7.2 ± 2.5 (1.0–12.8)	6.8 ± 2.4 (1.0–10.2)	7.4 ± 2.5 (1.5–12.8)	n.s.
Anteroposterior locking plate position, mm	11.5 ± 6.4 (− 1.4–25.2)	12.0 ± 7.7 (0.8–25.2)	11.3 ± 5.9 (− 1.4–23.6)	n.s.
Locking plate angle, °	29.6 ± 10.0 (3.0–52.4)	25.9 ± 10.0 (3.0–39.2)	31.1 ± 9.7 (8.6–52.4)	< 0.05

Data are presented as means ± standard deviation with the range in parentheses

## Discussion

The most important finding of this surgical simulation study was that the frequency of interference between screws and tibial tunnels differed depending on the tibial tunnel position. In previous studies, good clinical outcomes of transtibial pull-out repair with OWHTO were reported in knees with MMPRTs and varus malalignment [11, 15]. However, the relationship between locking screws and tibial tunnels has not been evaluated. To our knowledge, this is the first study evaluating the relationship between locking screws and each tibial tunnel in transtibial pull-out repair with OWHTO.

In the AM position, the frequency of interference between the tibial tunnel and screws B and C was higher than that of the PM and AL positions. Even if the plate position and tibial tunnel position were adjusted, complete prevention of the interference between screws and tibial tunnels seems impossible because the frequency of interference between the tibial tunnel and at least one screw in the AM position was 100% in this study. In addition, the locking screw should interfere with the tibial tunnel at the medial half of the tibial plateau because the tibial tunnel is made between the medial meniscus posterior root and medial tibial cortex. Thus, the screw length should be short to prevent interference, which may lead to weakness of the fixation stability in OWHTO.

The frequency of interference between screw A and the tibial tunnel in the AL position was higher than that in the AM and PM positions. The locking plate was placed more parallel to the medial/lateral axis of the tibial plateau in the interference group. In a previous study, locking screws were inserted longer towards the lateral tibial plateau by placing the locking plate in the medial direction [21]. This could lead interference between the tibial tunnel and locking screws at the lateral proximal tibia. Placing the locking plate in an anteromedial direction could be useful to prevent interference between locking screws and the tibial tunnel in the AL position. Meanwhile, there is a risk of damaging the tibial plateau when the position of the tibial tunnel is high. In this study, the mean height of the locking plate position was 7.2 mm and this seems to be a relatively high placement. Placing the locking plate more distally may reduce the frequency of interference with locking screws. An additional skin incision is also needed to make the tibial tunnel in the AL position.

In the PM position, only 7 knees (9.3%) interfered with screw C. The tibial tunnel did not interfere with screws A, B and D. The locking plate was placed more posteriorly in the group where the locking screw interfered with the tibial tunnel in the PM position. This is reasonable because the tibial tunnel was made between the medial meniscus posterior root and the posteromedial tibial cortex. Meanwhile, if screw C interferes with the tibial tunnel, the length of the screw should be short because screw C interferes with the tibial tunnel at the medial half of the tibial plateau. Anterior placement of the locking plate could be useful to prevent interference between locking screws and the tibial tunnel in the PM position.

Considering the results of the present study, making the tibial tunnel in the AM position should be avoided because interference between the tibial tunnel and locking screws was inevitable. When the tibial tunnel is created in the PM position, surgeons only need to pay attention to the interference between the tibial tunnel and screw C because screws A, B and D did not interfere with the tibial tunnel in the PM position in the present study. First, the tibial tunnel should be created in the PM position before the transverse and ascending cut. After opening the osteotomy site, a metal rod is inserted to the tibial tunnel. A Kirschner wire is inserted to the C hole for the temporary fixation of the locking plate and surgeons confirm that the Kirschner wire does not interfere with the metal rod in the tibial tunnel. The plate position should be adjusted when the Kirschner wire interferes with the metal rod in the tibial tunnel. When the tibial tunnel is created in the AL position, surgeons should pay attention to interference between the tibial tunnel and screws A, B and C because the tibial tunnel in the AL position interfered with these screws in this study. As in the case of the PM position, the tibial tunnel should be created in the AL position before the osteotomy. After opening the osteotomy site, a metal rod is inserted to the tibial tunnel and the temporary fixation of the locking plate with Kirschner wires was performed. The plate position should be adjusted so that Kirschner wires do not interfere with the metal rod in the tibial tunnel. It seems to be difficult to completely prevent the interference between locking screws and the tibial tunnel in the AL position because the tibial tunnel in the AL position interfered with screw A in 20% in this study. On the other hand, even if the screw A interfered with the tibial tunnel in the AL position, the screw could be inserted over the tibial plateau centre because the tibial tunnel was made between the medial meniscus posterior root and lateral tibial cortex. Thus, the effect of the shortening of the screw length on the fixation stability seems to be relatively small in the AL position.

Meanwhile, alteration of the locking plate position can affect the stability of the osteotomy site. In a previous biomechanical study, the anteromedial plate position led to the inferior stability of the osteotomy site without bone substitute compared to the medial plate position [28]. Bone substitute placement into the osteotomy site led to no difference of the stability between the anteromedial and medial plate positions [28]. Thus, when alteration the plate position to avoid interference between the tibial tunnel and locking screws, the use of bone substitute could be useful to compensate for the decreased stability of the osteotomy site.

In addition, good clinical outcomes of isolated OWHTOs for MMPRTs were reported in previous studies [7, 15, 16, 18, 22, 26]. Kim et al. [9] reported that the existence of MMPRTs did not affect the clinical outcomes of OWHTO in patients with varus knee OA and that repairing MMPRTs did not seem necessary during OWHTO. In a previous systematic review and meta-analysis, postoperative clinical outcomes and radiological and arthroscopic outcomes, except for the rate of complete meniscal healing, did not differ between OWHTO with repair of MMPRTs and isolated OWHTO during short-term follow-up [25]. Although long-term follow-up is needed to evaluate the effect of concomitant repair of MMPRTs with OWHTO, the necessity of concomitant pull-out repair of MMPRTs with OWHTO despite the risk of interference between the tibial tunnel and locking screws should be considered carefully.

There were a few limitations in this study. Because this study consists of a surgical simulation using CT images in patients after isolated OWHTO, the adjustment of the locking plate position for the tibial tunnel was not considered. The plate position could affect the results of this study. In addition, a tibial tunnel was created in such a way that the number of screws interfering with the tibial tunnel was minimized. If there were some patterns in which the number of interferences between the tibial tunnel and screws was the same, the tunnel position in which the longest screws could be inserted was chosen. This could have caused significant bias. Moreover, the attachment position of medial meniscus posterior root was determined based on CT images in this study. The tunnel was often difficult to be created at the real anatomical position with extruded meniscus. In that case, the tunnel can be a bit more anterior and medial compared to the anatomical position [4]. The relationship between the tibial tunnel and locking screws in patients who underwent concomitant transtibial pull-out repair with OWHTO should be elucidated in the future.

In daily clinical practice, making the tibial tunnel in the PM or AL position is recommended compared to the AM position because interference between the tibial tunnel in the AM position and locking screws was inevitable. When the tibial tunnel is created in the PM position, anterior placement of the locking plate is recommended to prevent interference between locking screws and the tibial tunnel. When the tibial tunnel is created in the AL position, placing the locking plate in an anteromedial direction is recommended to prevent interference between locking screws and the tibial tunnel.

## Conclusion

Making the tibial tunnel in the AM position should be avoided because interference with locking screws was inevitable. When the tibial tunnel is created in the PM position, interference between the tibial tunnel and screw C should be paid attention. Anterior placement of the locking plate could be useful to prevent interference between locking screws and the tibial tunnel in the PM position. In addition, when the tibial tunnel is created in the AL position, interference between the tibial tunnel and especially screw A among screws A–C should be paid attention. Placing the locking plate in an anteromedial direction could be useful to prevent interference between locking screws and the tibial tunnel in the AL position.

## Abbreviations

MMPRT: Medial meniscus posterior root tear
OWHTO: Open wedge high tibial osteotomy
OA: Osteoarthritis
CT: Computed tomography
AM: Anteromedial
PM: Posteromedial
AL: Anterolateral
